# Inflammation in chronic obstructive pulmonary disease and its role in cardiovascular disease and lung cancer

**DOI:** 10.1186/s40169-015-0068-z

**Published:** 2015-07-29

**Authors:** Paul T King

**Affiliations:** Monash Lung and Sleep, Monash Medical Centre, 246 Clayton Rd, Clayton, Melbourne, 3168 Australia; Monash University Department of Medicine, Monash Medical Centre, 246 Clayton Rd, Clayton, Melbourne, 3168 Australia

## Abstract

Chronic obstructive pulmonary disease (COPD) is characterized by lung inflammation that persists after smoking cessation. This inflammation is heterogeneous but the key inflammatory cell types involved are macrophages, neutrophils and T cells. Other lung cells may also produce inflammatory mediators, particularly the epithelial cells. The main inflammatory mediators include tumor necrosis factor alpha, interleukin-1, interleukin-6, reactive oxygen species and proteases. COPD is also associated with systemic inflammation and there is a markedly increased risk of cardiovascular disease (particularly coronary artery disease) and lung cancer in patients with COPD. There is strong associative evidence that the inflammatory cells/mediators in COPD are also relevant to the development of cardiovascular disease and lung cancer. There are a large number of potential inhibitors of inflammation in COPD that may well have beneficial effects for these comorbidities. This is a not well-understood area and there is a requirement for more definitive clinical and mechanistic studies to define the relationship between the inflammatory process of COPD and cardiovascular disease and lung cancer.

## Introduction

Chronic obstructive pulmonary disease (COPD) is characterized by chronic lung inflammation that results in progressive and irreversible airflow obstruction with periodic acute episodes of worsening, exacerbations. The airflow obstruction arises from a combination of emphysema and chronic bronchitis. It is predicted to be the third leading cause of death worldwide by 2020 [1], is a major cause of disability-adjusted life years (DALY) [2] and has a lifetime risk of up to 25% [3]. The inflammation in COPD is also systemic and this contributes to important comorbidities.

Smoking is the primary risk factor for COPD. However only 20–25% of smokers develop COPD. In addition once the inflammatory process in COPD is established it persists after smoking cessation [4, 5]. The inflammation is also associated with manifestations in addition to airflow obstruction, of which the two of the most important are cardiovascular disease (CVD) and lung cancer [6].

There is strong associative evidence that inflammatory process of COPD increases the risk of CVD and lung cancer but the mechanisms as to how this occurs are not well defined. This review will examine the relationship between the inflammation of COPD and CVD/lung cancer, and how this process could be potentially targeted therapeutically.

## The inflammatory process of COPD

The chronic inflammatory process in COPD involves both innate and adaptive immunity and is most pronounced in the bronchial walls of the small airways. The inflammatory process in COPD does have marked heterogeneity. It results in both emphysema with parenchymal involvement and chronic bronchitis, which predominantly affects the small airways. A characteristic feature of COPD is the presence of acute exacerbations, which are typically associated with increased inflammation. Important causes of exacerbations include infections (bacterial, viral and combined viral/bacteria) and environmental factors. Exacerbations of COPD are strongly associated with mortality, hospitalization and decline in functional status [7].

Smoking is the principal risk factor for COPD but biomass exposure particularly from cooking in poorly ventilated homes, is being increasingly recognized as being important [8]. Patients typically develop clinical symptoms many years after the initiation of smoking and this condition is generally diagnosed over the age of 50 years with a peak incidence at approximately 70 years [9].

Once established the inflammatory process in COPD is persistent despite smoking cessation and progresses over time [10]. It has been shown by Hogg et al. that after smoking cessation, there is progressive small airflow obstruction in patients with COPD, a number of years after smoking cessation. This small airflow obstruction was due to (1) the accumulation of inflammatory mucous exudates in the lumen and (2) increase in the tissue volume of the bronchial wall. The increase in the tissue volume of the bronchial wall was characterized by infiltration of the wall by both innate (macrophages/neutrophils) and adaptive inflammatory immune cells (CD4, CD8 and B lymphocytes) that formed lymphoid follicles.

The factors that drive inflammation in COPD after smoking cessation have not been clearly established although autoimmunity, embedded particles/heavy metals from smoking and chronic bacterial infection have all been proposed to have a role [11]. The most commonly associated factor with lung inflammation in COPD is autoimmunity. Lee et al. showed that emphysema is an autoimmune disease characterized by the presence of antielastin antibody and T-helper type 1 [T(H)1] responses, which correlates with emphysema severity [12]. Using both in vivo animal models and human lung epithelial cells Ghio et al. showed that cigarette smoking increased lung iron and ferritin levels [13]. A recent study described increased levels of cadmium and manganese in the lungs of patients with advanced COPD [14]. New work has highlighted the importance of the microbiome in lung disease [15–17]. The most common bacterium isolated from the lungs of patients with COPD is nontypeable *Haemophilus influenzae* (NTHi). NTHi induces changes of COPD in an animal model [18] and new strains are also associated with exacerbations of COPD [19]. This bacterium has also been shown to activate lung T cells [20] and cause the expression of reactive oxygen species and proteases in patients with COPD [21].

### Inflammatory cell types prominent in COPD

There are a variety of cell-types that contribute to inflammation in COPD, of which the most important are the macrophages, the neutrophils and the lymphocytes [11, 22, 23].

#### Macrophages

Macrophages have a key role in the pathogenesis of COPD and are found in markedly increased numbers in both the airways and lung parenchyma. There is a direct correlation between the presence of parenchymal macrophages and emphysema [24]. Smoking activates macrophages to produce a variety of inflammatory mediators including chemokines, reactive oxygen species (ROS) and proteases. Patients with COPD have macrophages that are more inflammatory than in subjects who smoke but who do not have COPD [25]. Macrophages are also heterogeneous and have different functional characteristics in different organs. These cells also display polarity in their function with M1 cells being more inflammatory and M2 cells being associated with a healing immune responses and fibrosis [26, 27]. Studies have reported that macrophages from patients with COPD have decreased phagocytosis of common bacteria such as *H. influenzae* and *Streptococcus pneumoniae* and this could be a contributing factor to chronic airway colonization [28–30].

#### Neutrophils

Neutrophils are generally more inflammatory than macrophages and are most prominent in acute exacerbations in the lung airways [23, 31]. Smoking increases the presence and activation of neutrophils in the respiratory tract [32]. Neutrophils are a potent source of proteases, particularly neutrophil elastase and reactive oxygen species (ROS).

#### Lymphocytes

As COPD progresses a distinctive feature is the development of lymphoid follicles in the small airway walls [10]. These lymphoid follicles are composed of T and B cells [33]. T cells are also found in the lung parenchyma and airways and studies have reported a relatively increased proportion of CD8+ cells to CD4+ cells. Studies have most commonly reported that these cells display a Th1/Tc1 polarity [20, 23, 32] although there is some variability in the literature whilst Th17 responses (defined by TH cells that produce the cytokine IL-17) have also been reported [34]. Roos et al. have recently shown that IL-17A is elevated in end-stage COPD and this contributes to cigarette smoke-induced lymphoid neogenesis [35]. Samples in this study were taken from human lung tissue and cigarette smoke exposed mice. Cigarette smoke exposure has been shown to upregulate IL-17 expression in human lung tissue [36] and in mice [37].

#### Other inflammatory cell types

Other inflammatory cell types involved in COPD include eosinophils, dendritic cells and mast cells. The presence of eosinophils in COPD may be associated with co-existent asthma and levels of eosinophil basic proteins have been found to be elevated as well [38]. Increased numbers of activated pulmonary dendritic cells are found in patients with COPD and are a marker of disease severity. Mast cells are widely distributed in the airways and have a well-established role in asthma inflammation [39]. In COPD mast cells upregulate markers of inflammation [40] and the distribution of these cells in the lung differs between centriacinar and panlobular emphysema [41].

### Structural lung cells and their contribution to lung inflammation

#### Epithelial cells

The epithelial cells also have an important role in mediating inflammation in COPD. These cells are activated by inhaled toxins, such as cigarette smoke and biomass smoke as well as by microorganisms [42]. This results in the production of a variety of inflammatory mediators including cytokine, chemokines and ROS. Smoking induces expression of the mediator CXCL14 by human epithelial cells and this is correlated with the development of lung cancer [43].

### Inflammatory mediators

There is a complex network of inflammatory mediators produced by inflammatory and structural cells in the lung including chemokines, growth factors and lipid mediators. The factors most clearly associated with pathogenic inflammation in COPD are cytokines, reactive oxygen species and proteases [11, 23]. The production of inflammatory mediators is triggered by the activation of Toll like receptors (TLRs) and lymphocyte antigen receptors which; through intracellular signaling pathways such as nuclear factor kappa-light-chain-enhancer of activated B cells (NF-κβ) and signal transducers and activators of transcription (STATs) leads to mediator release.

#### Cytokines

Cytokines in the lung are principally produced by macrophages and T cells. There is a range in the outcomes reported between different studies as a variety of different techniques have been used and studies have reported baseline levels and responses to stimulation. Important mediators produced by macrophages and neutrophils include tumor necrosis factor alpha (TNF-α), interleukin (IL)-1, IL-6 and IL-8. These cytokines are markedly proinflammatory in COPD [11, 23]. Consistent with Th1 responses, levels of interferon gamma (IFN-γ) and TNF-α are elevated in COPD particularly in the baseline state. These two cytokines are associated with active inflammation. Interestingly both IFN-γ and IL-13 (Th2) models have been associated with the development of emphysema in mice [44]. Recent publications have highlighted that there are elevated levels of IL-17 in patients with COPD [34].

#### Reactive oxygen species

The excessive production of reactive oxygen species damages the lung tissue and results in oxidative stress, which is a primary pathogenic process in COPD. The phagocytes (neutrophils and macrophages) and epithelial cells all produce ROS and this process is enhanced in patients with COPD. Antioxidants such as superoxide dismutase (SOD), glutathione and catalase are produced to counteract the effect of ROS and are regulated by the nuclear erythroid-2-related factor-2 (Nrf2) [45]. In patients with COPD there is deficient activation of Nrf2. ROS has a wide range of pro-inflammatory effects [46]. ROS also activates inflammatory transcription pathways [47]. Oxidative stress inhibits the activity of sirtuin-1 that is an important in tissue repair [48], and activates growth factors and damages DNA. Oxidative stress also contributes to accelerated aging of the lung in COPD [49].

#### Proteases

Proteases are produced by neutrophils and macrophages. These include neutrophil elastase and matrix metalloproteinases (MMP) 9 and 12. The proteases are principal factors driving the development of emphysema [50]. Their effect is opposed by anti-proteases such as α-1 antitrypsin. Deficiency of α-1 antitrypsin may result in the severe and early onset of emphysema. They also are involved with the activation of neutrophil-mediated inflammation [51] and increased arterial stiffness.

The binding of surface receptors (e.g. toll like receptors and antigen receptors) on immune cells by a diverse range of stimuli (including cytokines, ROS, oxidized low density lipoprotein and bacterial antigens) activates intracellular signaling pathways that drive the inflammatory response. Two important intracellular signaling pathways relevant to COPD are NF-κβ and STATs.

### Systemic inflammatory response

Chronic obstructive pulmonary disease is associated with a prominent systemic immune response that is more pronounced with advanced disease and in exacerbations. Systemic inflammation has been defined as the presence of inflammatory/immune response mediators that are present in the peripheral blood with levels that are elevated in COPD when compared to smoking controls (without COPD). Systemic inflammation as measured by the biomarkers; C-reactive protein (CRP), leukocytes and fibrinogen is associated with a two to four-fold increased risk of comorbidities including cardiovascular disease and lung cancer [52]. Another study used 6 inflammatory markers (CRP, IL-6, IL-8, TNF-α, fibrinogen and leukocytes) found elevation of at least one component in 70% of patients and persistent inflammation in 16%. Inflammatory biomarkers are also associated with cardiovascular disease and COPD and will be discussed in more detail in the individual sections below.

There have been a large number of studies that have described systemic inflammation occurring in COPD but the definition of what this entity is has often not been clear [53]. It is not clear if the systemic inflammatory response represents a spillover of mediators from the lung or is primarily a separate systemic component to the disease [54, 55]. This issue is of most relevance to complications such as osteoporosis, cerebrovascular disease and muscle wasting, rather than lung cancer and coronary artery disease with their intimate proximity to the lung.

## Cardiovascular disease and COPD

The risk of cardiovascular disease is significantly increased in patients with COPD. A diagnosis of COPD increases the risk of cardiovascular disease by an odds ratio (OR) of 2.7 [56]. COPD also increases the risk of mortality from CVD disease (OR of 1.68) [57]. There is marked overlap in risk factors in the two conditions particularly, smoking, older age and male sex. However lung inflammation is an important independent risk factor for cardiovascular disease and this section will discuss specific mechanisms involved in this process [58].

Inflammation has been increasingly recognized to have a role in atherosclerosis particularly in the context of coronary artery disease. Blood borne immune and inflammatory cells are an important component of atheroma. Macrophages and T cells infiltrate atheroma with the production of inflammatory cytokines. Activated immune cells (macrophages and T cells) are abundant at sites of plaque rupture and appear to play an important role in acute thrombosis and coronary syndrome [59, 60]. Important cytokines in this process include TNF-α, IFN- γ, IL-1 and IL-6.

### Coronary artery disease

In patients with COPD there is a significantly higher risk of coronary artery disease (OR 2.0), angina (OR 2.1) and myocardial infarction (OR 2.2) [56]. The degree of airflow obstruction is associated with increased mortality from ischemic heart disease [61]; for each drop of 10% in forced expiratory volume in 1 s (FEV1), CVD mortality increases by 28% and the incidence of non-fatal coronary events increases by 20% [62]. The presence of coronary artery disease is often not recognized. A study of inpatients with COPD found evidence of myocardial infarction on electrocardiogram in 27%, but in most subjects (70%) this was not documented in the clinical history [63].

The systemic inflammatory response may cause endothelial injury and vascular dysfunction, although the exact mechanisms of how this occurs remain to be determined. There are number of mechanisms which could contribute to this effect. Inflammatory cytokines produced by T cells and macrophages including IL-1, IL-6 and TNF-α all affect endothelial function [64]. There is increased endothelial permeability in COPD in response to mediators. The oxidative stress affects blood vessels including effects such as vasoconstriction and increased uptake of oxidated LDL cholesterol. There is inhibition of nitric oxide resulting in a vasoconstrictive state.

Patel et al. measured (1) arterial stiffness (as assessed by aortic wave velocity) and (2), cardiac biomarkers [troponin and N-terminal pro-brain natriuretic peptide (BNP)] and their relationship to COPD exacerbations and airway infection/inflammation [65]. They found that frequent COPD exacerbators had greater arterial stiffness than infrequent exacerbators and this was significantly associated with the inflammatory state as defined by the sputum levels of IL-6. In addition exacerbations of COPD were associated with significant rises in troponin and BNP, and the rises in these cardiac biomarkers correlated with higher serum CRP and sputum IL-8.

Thomsen et al. measured the relationship between elevated levels of three inflammatory biomarkers (CRP, fibrinogen, and leukocyte count) and comorbidities in a cohort of 8,656 patients with COPD [66]. They found that the risk of ischemic heart disease was increased by a factor of 2.19 in subjects who had all three biomarkers elevated (when compared to subjects who had all biomarkers in the normal range). Corresponding hazard ratios were 2.32 for myocardial infarction and 2.63 for heart failure.

The inflammation in COPD increases coagulopathy. Patients with this condition have elevated basal levels of Factor VIIa, tissue factor and thrombin-antithrombin complexes [67]. Plasma fibrin clots from patients with COPD are denser and more resistant to lysis and this effect can be inhibited by the use of simvastatin [68]. In addition hypoxaemia in patients with COPD, elevates levels of IL-6, prothrombin activation fragments and thrombin-antithrombin complex [69].

In unstable angina neutrophilic inflammation is present in the coronary arteries, suggesting that acute inflammation has a role in this process [58, 70]. It has recently been proposed that acute exacerbations of COPD with their associated neutrophil influx may be triggers for acute coronary events [71]. This proposal is supported by studies that have demonstrated that both acute respiratory infection and exacerbations of COPD are associated with a markedly increased incidence of acute coronary events [72, 73].

Troponin is a marker of myocardial stress/ischemia. Patients with COPD have elevated troponin levels, both at baseline and particularly in context of acute exacerbations [74, 75]. The elevation of troponin in the context of AECOPD is associated with the presence of neutrophilia consistent with an inflammatory component [76].

### Pulmonary artery disease

Changes in the pulmonary arteries occur in COPD and may result in pulmonary hypertension and right heart hypertrophy and this is an important factor in the increased risk of mortality. There are a variety of factors that contribute to this process, of which the two most important appear to be endothelial dysfunction [77] and coagulopathy. Hypoxaemia is recognized to have an important role in the development of pulmonary artery disease [78]. The relation between pulmonary artery disease and inflammation is less well defined than the previously discussed cardiac comorbidities. There is increased infiltration of pulmonary arteries by CD8+ T cells, which correlates with pulmonary hypertension. Other inflammatory markers associated with pulmonary artery disease in COPD include CRP, IL-6, MCP-1, TNF-α and phospholipid ceramide [79].

### Venous thromboembolism

There is an increased risk of venous thromboembolism (VTE) in patients with COPD. This is an area that has not been comprehensively studied, and there is a range in findings. In the context of acute exacerbations VTE is present in 3–29% of patients [80, 81]. Occult pulmonary embolism (PE) may have a role in precipitating AECOPD as well. There is an increased incidence of all the key risk factors for VTE in COPD (coagulopathy, endothelial dysfunction and venous stasis). The higher risk of VTE in exacerbations suggests that an inflammatory component may be relevant.

### Cerebrovascular disease

There appears to be a moderately increased risk of cerebrovascular disease in COPD, both in stable state and with acute exacerbations [82]. The risk is inversely proportional to the FEV1 and is more prominent in younger patients [83]. Systemic inflammation increases the risk of acute ischemic stroke [84]. Studies have reported worse neurological deficits in association with preceding infection in human studies and in animal models [85, 86]. Similar to coronary artery disease, activated macrophages and T cells are prominent in plaques in the cerebral circulation and secrete proteases such as MMPs which destabilize the plaque and increase the risk of rupture.

## Lung cancer and COPD

Lung cancer (defined as primary bronchogenic lung cancer) is the leading cause of cancer death worldwide, accounting for 1.59 million deaths in 2012 [87]. Smoking is the primary risk factor associated with lung cancer. However only 20–25% of smokers develop COPD and the presence of COPD is associated with a two to six times risk for the development of lung cancer compared to smokers without COPD [88, 89]. In addition COPD is associated with lung cancer in never smokers. The mortality of lung cancer is also correlated with the co-existence of COPD. The risk is highest for squamous cell lung carcinomas [89]. Airflow obstruction and the presence of emphysema on CT scanning are also independent risk factors for the development of lung cancer [89, 90].

The pathways that lead to the development of lung cancer are complex and only partially understood. In addition to smoking, chromosomal translocation and epigenetic modifications have important roles in the development of lung cancer [91–94]. Finally the primary pathogenesis of COPD is characterized by lung inflammation and there is increasing data about the role of inflammation in the development of cancer [95]. In a large prospective study an elevated CRP level increased the risk of lung cancer [96]. The use of inhaled corticosteroids have been associated the decreased risk of lung cancer although this study was retrospective [97]. The large variety of inflammatory mediators present in the lungs of COPD patients promotes the development of epithelial-to-mesenchymal transition (EMT) and lung cancer [98]. In animal models inflammation is a crucial factor in the development of lung cancer and depletion of macrophages and B cells is protective [32]. There are a number of inflammatory processes that can potentially contribute to the development of lung cancer and these will be discussed in more detail.

### Inflammatory cell types and lung cancer

Lung cancer is associated with an inflammatory environment with localized leukocyte infiltration and the most well characterized cellular responses have been of macrophages and T cells. Recent literature has also highlighted the role of other cell types such as the neutrophils and dendritic cells.

#### Macrophages

Monocytes migrate into tumors where they differentiate into macrophages under the influence of factors including monocyte-chemotactic protein (MCP-1), vascular endothelial growth factor (VEGF) and hypoxia. The differentiated macrophages in the tumor environment are designated as tumor associated macrophages (TAMs) [99]. These TAMs can develop into polarized M1 or M2 phenotypes. The M1 phenotype TAMs, are typically associated with antimicrobial function and tumor cell killing/inhibition. In contrast the M2 TAMs have a different cytokine functional profile and are associated with tissue remodeling/repair and angiogenesis; they are also tumor promoting [100]. In the early phase of tumor development the M-1 phenotype predominates whilst as tumor development progresses the M-2 phenotype becomes more dominant particularly in hypoxic tumor regions [101]. These hypoxic-driven TAMs express pro-angiogenic factors such as vascular endothelial growth factor (VEGF) and MMP9 as well as lymphangiogenic factors. The M1 and M2 responses are mutually antagonistic. Differentiating between M1 and M2 macrophages may be challenging and these cells demonstrate significant plasticity [102]. Published studies have been done predominantly in subjects with non small cell lung cancer (NSCLC).

#### Neutrophils

Neutrophils have been less intensively studied than macrophages in lung cancer. Several studies described the presence of tumor-associated neutrophils (TAN) in murine models. Eruslanov et al. have recently described that TANs have a prominent role in early-stage human lung cancer [103] and constituted between 5 and 25% of cells in these tumors. The TANs in this study displayed an activated phenotype with a distinct repertoire of chemokine receptors and proinflammatory factor production including MCP-1, IL-8, MIP1α and IL-6. This study concentrated on early stages of lung cancer and found that the TANs were associated with stimulation of T cell responses and could represent a type 1 TAN. Similar to M1/M2 polarization a paradigm of N1 (antitumor) and N2 (protumor) responses has been proposed in murine models of lung cancer [104].

#### Lymphocytes

Lymphocyte responses are important in cancer, in particular cytotoxic T cell responses have a key role in controlling/eliminating neoplastic cells. Advanced COPD is characterized by prominent infiltration of the lung by lymphocytes, particularly CD8+ cells [50]. The nature of the T cell responses has still not been well defined in COPD although the bulk of the published literature describes a Th1/Tc1 predominant response. Such a response would be expected to be proinflammatory with enhanced antitumor M1 responses. In contrast a study of responses of lung tissue T cells to stimulation by the bacterium *H. influenzae* found that subjects with COPD had a Th2/Tc2 predominant response [20]. However this area is still not well defined. Th17 cells appear to be relevant in the development of lung cancer and will be discussed in more detail in the subsequent section [105].

Other cell types also have a potential role in COPD and lung cancer, including natural (NK) cells, mast cells, dendritic cells and eosinophils [32, 106]. Defective NK cell responses may be important in the development of metastatic disease. There is conflicting published literature about the role of dendritic cells in lung cancer.

### Inflammatory mediators and lung cancer

There are a large number of inflammatory mediators produced in COPD, which are relevant for the development of lung cancer but their interactions are complex and not fully understood in this context.

#### Cytokines

Three prominent cytokines produced in the COPD are TNF-α, IL-1, and IL-6, and these all also have important roles in lung cancer. Tumor necrosis factor-α, which is predominantly secreted by T cells and macrophages, promotes carcinogenesis by inducing cell transformation/proliferation and tumor growth [107]. Malignant cells may also produce TNF-α, which may enhance tumor development. Interleukin-1 is produced by macrophages and tumor cells and is associated with enhanced tumorigenesis and the production of mediators such as ROS and proteases [108]. IL-1 and TNF-α are key mediators involved in the initiation of the inflammatory response. Interleukin 6 is secreted by macrophages and also by T cells (in the context of chronic inflammation) and has been clearly implicated both in the pathogenesis of COPD and in lung cancer. Ochoa et al. ablated IL-6 in a mouse cancer model and found that this markedly inhibited both the development of COPD-like lung inflammation and lung cancer [109]. IL-6 levels are elevated in lung cancer patients in cells lines with endothelial growth factor receptor (EGFR) mutations which results in STAT3 elevation [110].

Cytokines such as TNF-α, IL-1 and IL-6 induce transcription factors that drive inflammation including NF-κβ and STAT3. Both of these transcription factors/pathways are upregulated in COPD and lung cancer. There are a number of links between NF-κβ and carcinogenesis including upregulation of inflammation, cell transformation, proliferation and migration, and enhancement of the production of MMPs and angiogenic factors [111]. STAT3 is present in the cytoplasm and are activated by several factors particularly IL-6. STAT3 may upregulate expression of NF-κβ and has increased expression in lung cancer. The NF-κβ and STAT3 pathways facilitate the development of EMT [98, 112].

#### Proteases

Proteases such as neutrophil elastase and MMP9 and MMP12 have a key role in the pathogenesis of COPD. The MMPs have also been strongly implicated in the pathogenesis of lung cancer and have a variety of effects including the promotion of angiogenesis. The degradation of components of the extracellular matrix by proteases activates growth factors that stimulate endothelial cells to multiply and produce new capillaries, driving angiogenesis [113, 114]. Proteases may also facilitate metastasis by breaking down the lung matrix [115].

#### Reactive oxygen species

Oxidative stress with the excess production of reactive oxygen species is one the key pathogenic processes in COPD. Oxidative stress also has a major role in the development of lung cancer. ROS damages DNA and promotes its mutation by inhibiting the function of DNA repair genes and increases the expression of DNA methyltransferases [116]. As discussed previously the Nrf2 transcription factor may be downregulated in COPD with potentially enhanced damage from ROS.

#### Systemic inflammatory markers

Systemic inflammatory markers are also associated with an increased risk of lung cancer and there have been a number of publications in this area particularly in relation to CRP. Thomsen et al. found that the elevation of three inflammatory markers (CRP, fibrinogen and leukocyte count) had a hazard ratio of 4.0 for the development of lung cancer [66]. A study assessed the correlation between circulating inflammatory markers and the prospective development of lung cancer [117]. Significant associations were found between the risk of lung cancer and levels of both CRP and serum amyloid A protein. A meta-analysis of the association between CRP, IL-6 and lung cancer; found that CRP was associated with an increased risk of lung cancer (particularly in men) but there was no association with Il-6 levels [118].

Table 1 summarizes the key inflammatory cells/mediators and their effects in COPD, CVD and lung cancer.

**Table 1:** Common inflammatory cells/mediators in COPD, CVD and lung cancer

	COPD	CVD	Lung cancer
Inflammatory cell			
Macrophage	Primary producer of inflammatory mediators in COPD, M1/M2 function	Major producer of inflammatory mediators, important in plaque rupture	Primary inflammatory cell in lung cancer, TAM1/TAM2 function
Neutrophil	Key role in exacerbations, produces IL-8, elastase	May have role in precipitating acute coronary events	Proinflammatory, produces IL-8, elastase
T cell	Key driver of inflammation in advanced disease, major source of cytokines	Infiltration of plaques	CD8+ cells important in killing tumor cells, secretor of cytokines
Inflammatory mediator			
TNF-α	Marked proinflammatory effect	Proinflammatory, affects endothelial function	Proinflammatory, promotes carcinogenesis
IL-1	Proinflammatory effect	Proinflammatory, affects endothelial function	Enhances tumorigenesis and ROS/proteases
IL-6	Proinflammatory effect	Proinflammatory affects endothelial function	Enhances tumorigenesis (STAT3), inflammation
ROS	Damages lung tissue	Proinflammatory, damages endothelium	Damages DNA, enhances tumorigenesis
Proteases	Causes emphysema	Role in plaque instability	Promote tumor angiogenesis/metastasis

*COPD* chronic obstructive pulmonary disease, *CVD* cardiovascular disease, *M1/M2* types one and two macrophage subsets, *TAM* tumor-associated macrophages, *IL* interleukin, *TNF-α* tumor necrosis factor alpha, *STAT* signal transducers and activators of transcription, *ROS* reactive oxygen species.

## Therapeutic strategies against COPD to reduce CVD and lung cancer

There has been extensive interest over many years in agents that could be used to reduce inflammation in COPD. The reduction of inflammation in COPD may reduce the increased risk of CVD and lung cancer that occurs in this context, although the clinical trials that have addressed this issue are very limited.

**Fig. 1:**
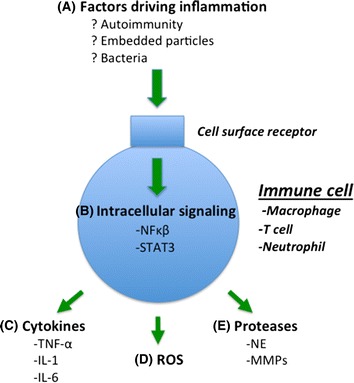
Potential targets for anti-inflammatory therapy The inflammatory process in COPD is associated with cardiovascular disease and lung cancer and is a potential therapeutic target to reduce these comorbidities. In additional to broad spectrum therapies such as corticosteroids specific anitinflammatory therapies could be directed towards **a** the primary factors driving inflammation in COPD, **b** intracellular signaling pathways including NF-κβ and STAT3 e.g. by tyrosine kinase inhibitors, **c** inhibition of specific inflammatory cytokines such as tumor necrosis factor alpha (TNF-α) and interleukins (IL) 1 and 6 and the generation of **d** reactive oxygen species (ROS) and **e** proteases such as neutrophil elastase (NE) and matrix metalloproteinases 9 and 12 (MMP9/12).

Pharmaceutical therapy for the treatment of inflammatory process in COPD is relatively ineffective [119]. The major therapy that has been used is corticosteroids both in systemic form and particularly as inhalational therapy. Whilst inhaled corticosteroids (ICS) reduce frequency of exacerbations in COPD most patients are resistant to these medications for the control of chronic inflammation. However a retrospective study reported that the use of ICS reduced lung cancer [97]. Corticosteroids have very broad immunosuppressive effects and particularly in long-term use, major side effects. Recent work has concentrated on developing more targeted approaches to immune suppression in COPD. In this section the work that is most relevant to the comorbidities of CVD and lung cancer will be summarized. Potential key targets are highlighted in Fig. 1.

### Therapies to target potential causes of lung inflammation

The most important therapeutic intervention in the treatment of COPD is the cessation of smoking. However inflammation in COPD persists, and in more advanced disease progresses despite smoking cessation. This persistence of the inflammatory process after smoking cessation is not well understood but represents a potential major therapeutic target. There have been studies demonstrating that autoimmunity may have a role in the pathogenesis of COPD but standard anti-inflammatory therapies have so far been relatively ineffective. Bacterial infection is very prevalent in COPD and antibiotics have been used as standard therapy for exacerbations. The role of bacteria in the chronic inflammatory process of COPD is still not well defined. There have been a number of recent trials of macrolides as maintenance therapy, which have demonstrated a marked reduction in the frequency of exacerbations of COPD; whether this is antimicrobial or anti-inflammatory effect is not clear [120]. A recent trial of anti-IL-17 therapy found there was a reduction in lung cancer in a mouse model and this also occurred with *H. influenzae* infection [105].

### Intracellular signaling pathways

The primary stimuli act on the cell surface receptors (e.g. toll like receptors and lymphocyte receptors) to activate intracellular signaling pathways to drive the production of inflammatory mediators. These signaling pathways are complex but tyrosine kinases are major drivers of this cascade and activate downstream pathways such as NF-κβ and STAT3. Tyrosine kinase inhibitors include phosphodiesterase inhibitors that act to inhibit intracellular cyclic AMP and GMP, with a variety of effects including inhibition of inflammatory mediators. Theophylline is a phosphodiesterase inhibitor that has been used for many years but only has mild therapeutic effects with a high incidence of toxicity. The phosphodiesterase inhibitor roflumilast inhibits neutrophil recruitment and activation, T cell activation and monocyte chemotaxis and has clinical benefits including reducing exacerbations and improving lung function although it did not alter levels of CRP [121–123]. Roflumilast does have a high incidence of side effects though. Kinase inhibitors affect important inflammatory signaling pathways including NF-κβ and STAT3 production and the production of inflammatory mediators such as IL-1, TNF-α and MMP9. There are number of kinase inhibitors that have been developed particularly against p38 mitogen-activated protein kinase and phosphoinositide 3-kinase but these compounds are currently mainly being tested in the pre-clinical setting.

### Cytokine production

The inflammatory cytokines, TNF-α, IL-1 and IL-6 are elevated in COPD, CVD, and lung cancer and are potential therapeutic targets particularly in the context of acute exacerbations. There are several strategies that can be used to inhibit the effects of IL-1 and these include receptor antagonists, blocking monoclonal antibodies and an IL-1 trap. These approaches have been demonstrated useful effects in other inflammatory diseases such as gout [124]. Currenlty an IL-1 blocking antibody (canakinumab) is being investigated for the treatment of several conditions including COPD [125]. TNF-α has a major role in driving inflammation and targeted therapies in rheumatoid arthritis with blocking antibody therapy such as infliximab have been highly effective. Unfortunately infliximab in a trial in patients with COPD showed no clinical improvement after 6 months and was associated with major side effects [126]. Anti IL-6 antibodies (tocilizumab) have benefits in patients with refractory rheumatoid arthritis but have not been tried in patients with COPD [127]. Interleukin 8 is a key chemotactic factor for neutrophils and monocytes but a clinical trial of a blocking antibody failed to show benefit [128]. An issue for the treatment of COPD-driven inflammation and its associated comorbidities is that there is elevation of many inflammatory mediators and the targeting of one specific cytokine has so far proved ineffective.

#### Protease and reactive oxygen species production

Two broad inflammatory processes relevant to COPD are protease imbalance and oxidative stress. There are specific anti-proteases available. A specific inhibitor of neutrophil elastase is effective in an animal model but was not found to be beneficial in a clinical trail [129]. The MMPs may be more important in the chronic inflammatory process than neutrophil elastase, but a dual MMP9/MMP12 inhibitor developed in animals has not yet been used in clinical trials at this stage [130]. Oxidative stress has been a therapeutic target in a variety of conditions but has generally been relatively resistant. *N*-acetylcysteine is a well-established therapy but its anti-oxidant effect has generally been disappointing. A number of new and more potent anti-oxidants have been developed but in limited animal and clinical trials have generally not been effective.

### Corticosteroids

Corticosteroids remain the cornerstone of asthma treatment but are generally relatively ineffective in COPD. There has been renewed interest in the identification of factors that could reverse this corticosteroid resistance. A potential mechanism is reduced histone deacetylase 2-mediated deacetylation of the glucocorticoid receptor expression with associated oxidative stress [131]. The addition of other agents with corticosteroids such as low-dose theophylline and tricyclic antidepressants may be potentially beneficial in this circumstance [132, 133].

### Statins

Statins are a potentially promising therapy in COPD. In addition to their lipid lowering properties they reduce the stability of lipid raft formation with inhibition of signaling and cytokine, chemokine and adhesion molecule expression. This inhibits systemic inflammation with decreased CRP. Two systematic reviews of the observational studies published have reported beneficial effects of statins in COPD, including decreased mortality and improved lung function [134, 135]. Statins may also have a role in improving lung cancer outcomes. A large retrospective study reported that the used of statins reduced lung cancer mortality and metastatic disease [136].

## Conclusions and future directions

Chronic obstructive pulmonary disease is characterized by lung inflammation that persists after smoking cessation. This inflammation is heterogeneous but the key cell types involved are macrophages, neutrophils and T cells, The dominant inflammatory mediators include TNF-α, IL-1, IL-6, reactive oxygen species and proteases. COPD is also associated with systemic inflammation and there is a markedly increased risk of cardiovascular disease (particularly coronary artery disease) and lung cancer in patients with COPD. This inflammation is an independent risk factor (additional to smoking, male sex etc.) for these comorbidities. There is strong associative evidence that the inflammatory cells/mediators in COPD are also relevant to the development of CVD and lung cancer. There are a large number of potential inhibitors of inflammation in COPD that may well have beneficial effects for these comorbidities. Particularly in lung cancer, which has a 5-year survival of less than 20%, the development of new and more effective therapies is urgently needed.

There are some priority areas in the study of COPD and its associated CVD and lung cancer morbidities (see Table 2). Firstly, there needs to be more definitive human clinical studies to establish the link between COPD and CVD/lung cancer, particularly large prospective trials. The mechanisms that link COPD with its comorbidities have not been adequately defined due to lack of established experimental/animal models. Finally there is a need for the development of targeted inhibitors of inflammation, which prevent harmful immune responses but do not have major side effects. Perhaps the most potentially useful area to target is to identify the specific factors that drive inflammation in COPD after smoking cessation.

**Table 2:** Priority areas

More definitive, prospective, human clinical studies to define the links between the inflammatory process of COPD and cardiovascular disease/lung cancer	
The development of experimental/animal models to define how the inflammation in COPD increases the risk of cardiovascular disease/lung cancer	
The development of targeted antiinflammatory therapies in COPD to reduce the associated cardiovascular disease/lung cancer	

## Abbreviations

BNP: N-terminal pro-brain natriuretic peptide
COPD: chronic obstructive pulmonary disease
CVD: cardiovascular disease
CRP: C-reactive protein
EGFR: endothelial growth factor receptor
EMT: epithelial-to-mesenchymal transition
IFN-γ: interferon gamma
IL: interleukin
MMP: matrix metalloproteinases
MCP-1: monocyte-chemotactic protein
NE: neutrophil elastase
NF-κβ: nuclear factor kappa-light-chain-enhancer of activated B cells
NK: natural killer
Nrf2: nuclear erythroid-2-related factor-2
NSCLC: non small cell lung cancer
NTHi: nontypeable *Haemophilus influenzae*
ROS: reactive oxygen species
STAT: signal transducers and activators of transcription
SOD: superoxide dismutase
TAMs: tumor associated macrophages
TAN: tumor-associated neutrophils
T(H)1: T-helper type 1
TNF-α: tumor necrosis factor alpha
VEGF: vascular endothelial growth factor
VTE: venous thromboembolism
